# A 25-year evolution of systemic mastocytosis to mast cell leukemia: Cutaneous clues

**DOI:** 10.1016/j.jdcr.2026.05.043

**Published:** 2026-05-26

**Authors:** Islam Younis, Brandy Ree, Sara C. Shalin, Hayley Creath, Mildred Clifton

**Affiliations:** a Department of Molecular and Cellular Biology, Arkansas College of Osteopathic Medicine, Fort Smith, Arkansas; b Department of Dermatology, University of Arkansas for Medical Sciences, Little Rock, Arkansas; c Premier Dermatology and Aesthetics, Bentonville, Arkansas

**Keywords:** avapritinib, hematodermatology, KIT mutation, leukemic transformation, maculopapular cutaneous mastocytosis (MPCM), mast cell leukemia (MCL), systemic mastocytosis, telangiectasia macularis eruptiva perstans (TMEP)

## Introduction

Systemic mastocytosis is a rare clonal neoplastic proliferation of mast cells characterized by diverse skin and systemic manifestations. These varied clinical signs and symptoms can contribute to delays in medical care and diagnosis. We report a case of a previously healthy man with a 25-year history of skin lesions consistent with telangiectasia macularis eruptiva perstans, later progressing to mast cell leukemia. This case highlights the diagnostic challenges of mast cell disease and the importance of early recognition and consideration of targeted therapy in advanced cases.

## Case report

**Fig 1:**
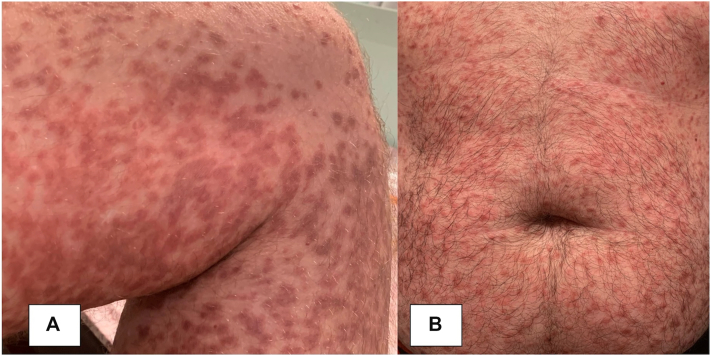
Telangiectasia macularis eruptive perstans; photographs showing widespread symmetric red-brown telangiectatic macules and thin papules, with focal confluence on the proximal lower extremity **(A)** and abdomen **(B)**.

A 52-year-old male presented with a 25-year history of persistent, intermittently pruritic, red-brown macules on the lower extremities, which eventually spread to the trunk and upper extremities (Fig 1). For over a decade, the eruption was misattributed to environmental triggers and behavioral causes. The patient reported chronic symptoms of mast cell mediator release, including abdominal pain, near-syncope, cognitive dysfunction (“brain fog”), and arthralgias, exacerbated by heat, stress, and spicy foods.

**Fig 2:**
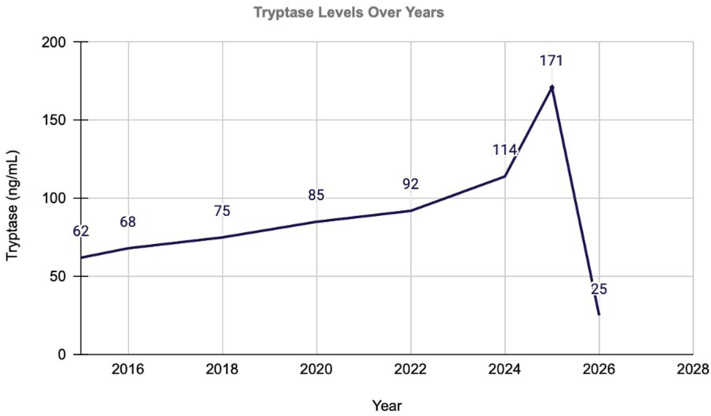
Serial serum tryptase levels of the index patient, 2014-2026. Levels were elevated at presentation (62 ng/mL), then rose progressively to a peak of 171 ng/mL in 2025, and declined to 25 ng/mL in 2026 after intervention with a small daily dose of avapritinib (25 mg).

In late 2014, a dermatology evaluation revealed widespread telangiectatic macules, which raised concern for maculopapular cutaneous mastocytosis, specifically, telangiectatic macularis eruptiva perstans. Skin punch biopsy demonstrated mast cells in the papillary dermis with oval nuclei and abundant eosinophilic cytoplasm. Serum tryptase was elevated at 62 ng/mL (normal: 1-15 ng/mL). A bone marrow (BM) biopsy performed in 2015 confirmed indolent systemic mastocytosis by fulfilling the diagnostic criteria set by the National Comprehensive Cancer Network. The patient showed no “C-findings”–such as cytopenias, palpable hepatosplenomegaly with functional impairment or malabsorption–and Sanger sequencing tests for *KIT* mutations were negative. Medical management varied over the following decade (See Table I) as his condition remained overall stable, with persistently elevated tryptase levels (Fig 2).

**Table I:** Progression of medical management of the patient during 2014-2024

Date	Management	Clinical notes
December 2014	Epinephrine 0.3 mg autoinjector	Prescribed to use as needed due to a high risk of anaphylaxis
2015	Cetirizine, fexofenadine, and hydroxyzine 25 mg tacrolimus 0.1% & hydrocortisone	Antihistamines were used for symptomatic control of pruritus and rash. Topical steroids were used in short, alternating courses with tacrolimus to prevent steroid atrophy
2020	Diphenhydramine 25 mg daily	Added to the regimen for further symptomatic control of the patient's cutaneous flares
May 2022	Naproxen	Instructed to take as needed with food to manage flares of generalized arthralgias and myalgias (bone/joint pain)
November 2022	Prednisone (Prolonged taper)	Prescribed to manage a severe systemic mastocytosis flare, starting at 80 mg and gradually walking down over several weeks
January 2024	Vitamin D3 (50,000 units) weekly methylprednisolone 4 mg	Vitamin D3 was prescribed to treat a documented deficiency. The patient also kept a methylprednisolone 4 mg at home for acute unforeseen symptom events
April 2024	Meloxicam 15 mg quercetin, diamine oxidase, and Low-histamine diet	Meloxicam was initiated for escalating bone pain. Instead of starting high-dose steroids for worsening symptoms and rising tryptase, the patient opted for dietary changes, quercetin, and diamine oxidase supplementation
June/July 2024	Omeprazole 40 mg Ketorolac 40 mg	Omeprazole replaced OTC omeprazole for GI symptoms. A trial of Ketorolac was started to manage escalating bone pain, replacing previous NSAIDs.
September 2024	Montelukast 5 mg Fenoxfenadine 180 mg	The patient discontinued night-time Benadryl and added Montelukast and an additional dose of Fenoxfenadine at bedtime to manage symptoms during cooler weather better

Table I outlines the decade-long progression of therapeutic interventions for a patient with systemic mastocytosis. It highlights key supportive care measures before escalation to tyrosine kinase inhibitors.

In the summer of 2020, his course was complicated by concomitant Rocky Mountain spotted fever and COVID-19 infections, both of which were associated with significant systemic inflammatory flares consisting of pruritus, arthralgias, myalgias, anxiety, and abdominal pain. In 2022, a repeat BM biopsy confirmed systemic mastocytosis with roughly 10% to 15% mast cell cellularity, favoring the indolent form, and positron emission tomography/computed tomography revealed widespread sclerotic bone foci. A repeat flow cytometry test detecting *KIT* mutations was negative.

By early 2024, the patient developed worsening exercise intolerance, near-syncope, palpitations, and severe episodic panic attacks, prompting urgent reassessment. Skin examination revealed stable lesions, with no new lesions detected. However, the tryptase levels had risen to 114 ng/ml. In May 2025, a next-generation sequencing test showed a negative *KIT* mutation, and a BM biopsy showed 90% hypercellularity with 20% to 30% mast cells, meeting the WHO diagnostic criteria^1^ for mast cell leukemia. An initial trial of imatinib (up to 400 mg daily) was discontinued due to debilitating side effects.

**Fig 3:**
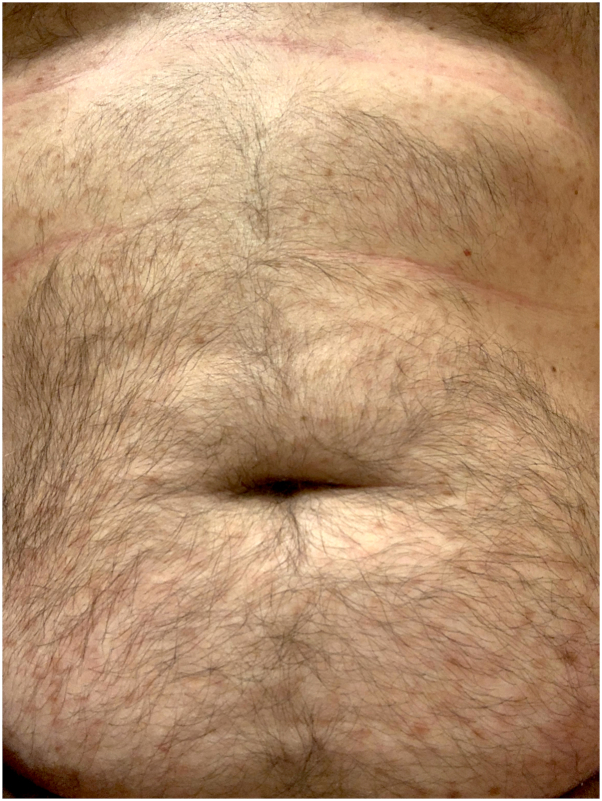
Clinical response following avapritinib therapy. The patient’s trunk and abdomen four months after initiation of avapritinib (25 mg daily). The image demonstrates near-complete resolution of the previously persistent, widespread telangiectatic macules characteristic of his systemic mastocytosis correlating with the patient's clinical improvement and the significant reduction in mast cell burden observed on the follow-up bone marrow biopsy.

By late 2025, tryptase peaked at 171 ng/mL. By late 2025, tryptase peaked at 171 ng/mL. A *KIT* mutation was eventually identified via High-sensitivity droplet digital PCR. A subsequent Droplet PCR test in February 2026 was also positive, with a quantitative value of **0.25%**. While the exact variant detected in 2025 is not detailed, the background clinical information from his earlier lab reports indicates that these diagnostic tests typically analyze ***KIT* exons 8, 9, 11, and 17**. The lab documentation specifically notes that the ***KIT* exon 17 D816V** mutation is commonly reported in patients with systemic mastocytosis. The patient was started on 25 mg of avapritinib daily (vs typical 200 mg daily) –due to concerns of medication sensitivity and tolerability. Within 4 months, a follow-up BM biopsy showed a dramatic reduction in mast cell burden to 5%, and tryptase plummeted to 25 ng/mL. The patient reported significant symptomatic improvement and remains on therapy. Finally, his lesions improved markedly, with near-resolution on his trunk and back, leaving only persistence on the lower extremities. He reported no pruritus (Fig 3).

## Discussion

This case highlights 3 main issues relevant to dermatologists:

1. Adult mastocytosis may present with subtle cutaneous findings and prolonged diagnostic delay. Telangiectasia macularis eruptiva perstans-like lesions–now recognized as a variant of maculopapular cutaneous mastocytosis^1^^,^^2^– may be overlooked, particularly when the ***Darier sign*** (localized urtication and erythema induced by rubbing a lesion) is absent or minimal. Recognition of widespread telangiectatic macules, with or without mediator-related symptoms (eg, pruritus, abdominal pain, hypotension, cognitive dysfunction, “brain fog”) should prompt a systemic workup for mastocytosis, including a bone marrow biopsy.^2^, ^3^, ^4^, ^5^
2. Negative initial molecular testing does not rule out systemic mastocytosis. While the majority of systemic mastocytosis cases document a *KIT* mutation, most often *KIT* D816V, detection of this mutation is not required for diagnosis, although it does represent a minor criterion.^6^ Mutations can be missed by low-sensitivity tests such as Sanger Sequencing, especially when the allele burden is low.^7^ More sensitive molecular methods can find *KIT* D816V in over 80% to 90% of patients with suspected systemic mastocytosis; on the other hand, about 5% to 10% of patients may test negative for *KIT* D816V, which highlights the risk of false negatives with less sensitive tests. For patients with compatible clinical signs, elevated tryptase levels, and supportive tissue analysis, repeat testing with a high-sensitivity method may be necessary.^4^^,^^8^
3. Serial serum tryptase measurements were clinically informative but not in isolation. Although elevated baseline tryptase may reflect mast cell burden, a marked change from baseline, along with objective clinical findings such as cytopenias, hepatosplenomegaly, liver dysfunction, ascites, elevated alkaline phosphatase, and pathological fractures, may indicate an increasing mast cell burden and should prompt reassessment in symptomatic patients.^3^^,^^4^^,^^8^ In this patient, the marked increase from baseline coincided with worsening clinical disease and subsequent leukemic transformation. A long-standing baseline range of ∼ 50 to 100 ng/mL was followed by a rise to 171 ng/mL at the time of leukemic transformation and coincided with reports of bone pain. The KIT inhibitor avapritinib elicited a rapid biochemical response, consistent with published trial data reporting high response rates in advanced systemic mastocytosis.^9^^,^^10^

## Conflicts of interest

None disclosed.
